# Reduced graphene oxide triggered epithelial-mesenchymal transition in A549 cells

**DOI:** 10.1038/s41598-018-33414-x

**Published:** 2018-10-12

**Authors:** Yanyan Liao, Weiyi Wang, Xiaomei Huang, Yongyan Sun, Shen Tian, Peng Cai

**Affiliations:** 1 Key Lab of Urban Environment and Health, Institute of Urban Environment, Chinese Academy of Sciences, Xiamen, 361021 China; 2 Xiamen Key Laboratory of Physical Environment, Institute of Urban Environment, Chinese Academy of Sciences, Xiamen, 361021 China; 3 College of Resources and Environment, University of Chinese Academy of Sciences, Beijing, 100049 China; 4 State Key Laboratory Breeding Base of Marine Genetic Resources, Key Laboratory of Marine Genetic Resources, Fujian Key Laboratory of Marine Genetic Resources, Fujian Collaborative Innovation Centre for Exploitation and Utilization of Marine Biological Resources, Third Institute of Oceanography, State Oceanic Administration, Xiamen, 361005 China; 5 Shanghai Institutes for Biological Sciences, Chinese Academy of Sciences, Shanghai, 200031 China

## Abstract

Graphene and its derivatives have exhibited wide potential applications in electronics, structural engineering and medicine. However, over utilization and untreated discharge may cause its distribution into environmental as well as biological chain, which raised the concerns of potential health risk as a potential hazard. Accumulating evidence has demonstrated that graphene derivatives induce lung fibrosis *in vivo*, so overall goal of this study was to explore the molecular mechanisms underlying the pulmonary fibrotic responses of reduced graphene oxide (rGO), using *in vitro* assays. Epithelial-mesenchymal transition (EMT) has profound effect on development of pulmonary fibrosis. Herein, we evaluated the EMT effect of rGO samples on A549 cells. Firstly, rGO penetrated through the A549 cells membrane into the cytosol by endocytosis and located in late endosome and/or lysosomes observed via transmission electron microscopy (TEM), and were well tolerant by cells. Secondly, rGO promoted the cell migration and invasion capacities at lower doses (below 10 μg/ml), but significantly inhibited the capacities at 20 μg/ml. Moreover, rGO-induced EMT were evidenced by decreased expression of epithelial marker like E-cadherin, β-catenin, Smad4 and increased expression of mesenchymal markers like Vimentin, VEGF-B, TWIST1. Based on our findings, it is supposed that rGO can effectively induce EMT through altering epithelial–mesenchymal transition markers in A549 cells.

## Introduction

Graphene is defined as a single-atom-thick sheet of monocrystalline graphite with sp^2^-bonded carbon atoms packed densely in a two-dimensional(2D) honeycomb lattice network. Since its discovery in 2004, grapheme and its derivatives have revealed attractive applications in many fields, including electrochemical devices, fluorescence imaging probes, gene/drug delivery, tissue engineering, cancer therapy, bacterial inhibition, and so on^1–5^, for their unique electronic and mechanical properties, superior electrical and thermal conductivity, a high surface to volume ratio and extraordinary mobility of charge carriers^6^.

In lieu of the great enthusiasm behind the potential application of graphene concurrently evoke the concern on their potential environmental health and safety influences. Therefore, prior to any prospective applications of graphene, it is imperative to assess their potential toxic effects, which is almost completely unknown compared with that of other carbon nanostructures, such as carbon nanotubes. Recently, a number of studies have tested its toxicity *in vitro* and *in vivo*, but the potential hazard of this nanomaterial is still obscure because toxic and nontoxic effects were simultaneously observed. These discrepancies may be due to differences in physicochemical properties including chemical composition, lateral dimension, surface area, shape, purity, functional groups, charges, coatings, dissolving media etc. Several studies demonstrated that graphene was nontoxic and biocompatible for biomedical applications at least under the limited condition^7–10^. Nevertheless, there are numerous studies demonstrated the *in vitro* and *in vivo* toxicity of graphene in different cell lines and animal models^7,11,12^. Mechanisms that were supposed to underlie the cytotoxic effect was reported as generation of reactive oxygen species (ROS) resulting in oxidative stress^13–16^, mitochondrial injury^14,16^, plasma membrane damage^16–19^, programmed cell death (apoptosis, autophagy, and programmed necrosis)^14,16,19–21^, immune responses^21^ and so on. In toxicity studies on graphene in laboratory animals, graphene induced potential pulmonary, systemic, behavioral, reproductive, and developmental toxicity and genotoxicity. Various studies showed that graphene induced only minimal pulmonary toxicity by inhalation exposure, whereas it caused acute and subacute pulmonary inflammation by bolus airway exposure^11^. Besides, fibrotic reactions or granulomas in the lungs of rats or mice were also observed following inhalation, intratracheal instillation and pharyngeal aspiration of graphene^22–24^. Although such studies revealed the pulmonary fibrotic responses is the adverse pathologic outcome after exposured to graphene nanomaterials, few studies were carried out to reveal the cellular and molecular mechanisms of pulmonary fibrosis exerted by graphene.

Epithelial–mesenchymal transition (EMT) is the gradual loss of epithelial cell polarity and the acquisition of mesenchymal characteristics that occurs during both development and disease, such as embryonic development, tissue fibrosis, tumor development and so on^25^. During this unique process, epithelial cells lose cellular polarity and cell–cell adhesion contacts, as well as increased motility, invasiveness, anti-apoptosis and production of extracellular matrix (ECM) components^26^. After the activation of the EMT program, the expression of polarized epithelial markers, such as E-cadherin, β-catenin and some cytokeratins, lost whereas mesenchymal markers, including vimentin, N-cadherin or of myofibroblasts, as α-smooth muscle actin (α-SMA) turn on. Although EMT was first noted during embryonic development and wound repair in normal tissues, it is increasingly acknowledged that EMT is an important pathway in fibrosis: differentiated epithelial cells undergo transition to a mesenchymal phenotype, giving rise to fibroblasts and myofibroblasts generation^27^. Moreover, several investigators reported that carbon nanotubes can promote lung fibrosis through EMT in human A549 cells and in rat alveolar type-II epithelial cells^28–30^. Graphene is the latest member of carbon nanomaterial, to improve our knowledge about the molecular mechanisms underlying graphene-induced toxicity, we elucidate the role of EMT in A549 (adenocarcinomic human alveolar basal epithelial) cells when exposed to reduced graphene oxide (rGO).

In this current study, we indicated for the first time that the rGO triggered EMT activation in A549 cells through a mechanism that involves epithelial markers downregulation and mesenchymal phenotype markers upregulation, and increased cell migration and invasion abilities. These results further highlight the possible adverse health effect caused by rGO exposure and enabled us to deeply understand the cellular and molecular mechanisms involved in rGO-induced pulmonary fibrosis.

## Results

### Characterization of rGO

**Figure 1:**
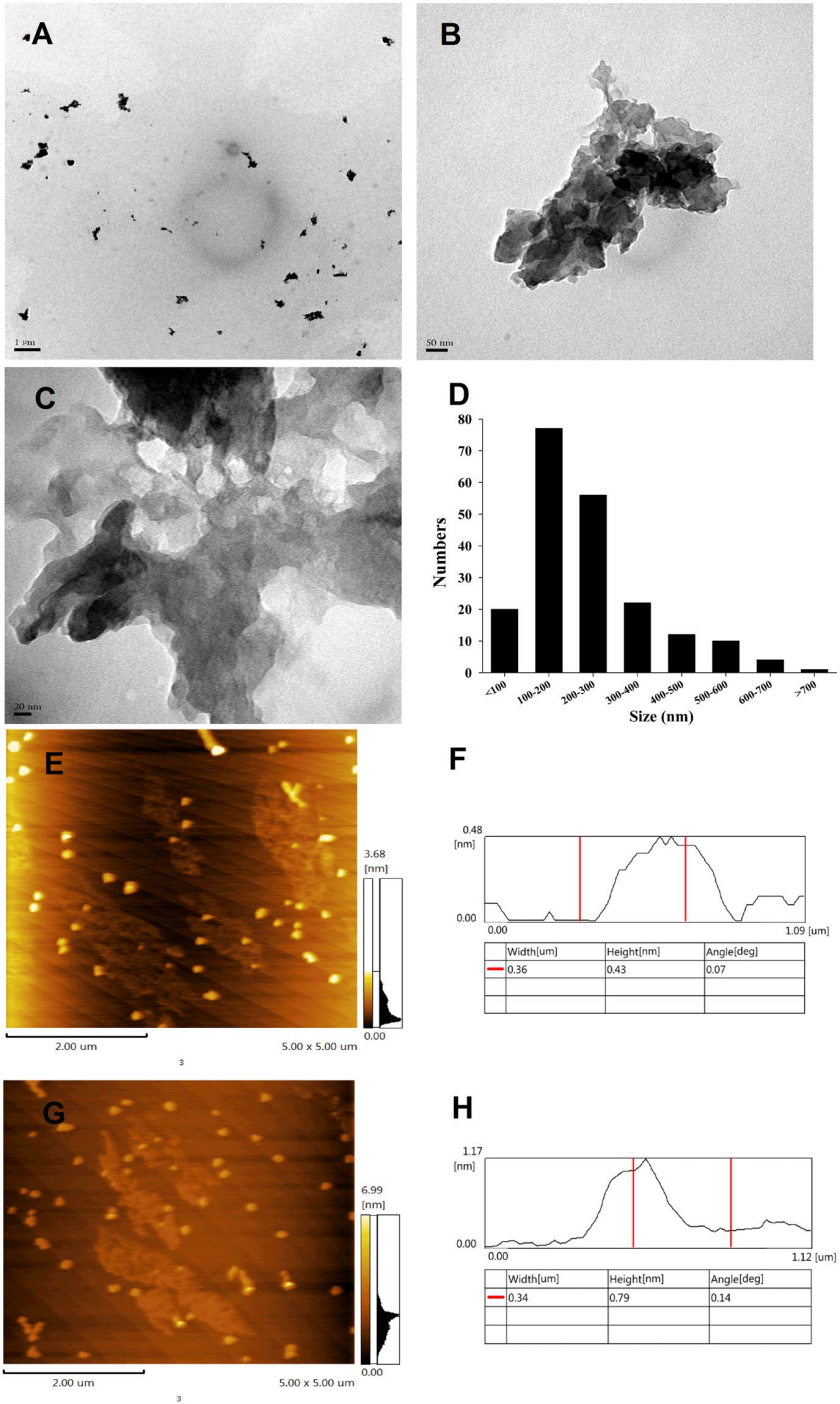
Characterization of the rGO after sonication. (**A**,**B**,**C**) TEM images. (**D**) size distributions of rGO. (**E**,**G**) AFM images. (**F**,**H**)AFM results of flake thickness for rGO.

rGO have been known to be in agglomerated nanosheets in aqueous suspension^31–33^. Similarly to those reported previously^34^, rGO nanosheets after sonication in current study also present as obviously wrinkled and scrolled structures (Fig. 1B,C). rGO nanosheets were either displayed individual particles or in the form of particle aggregates and agglomerates (Fig. 1A). TEM was used to analyze its surface morphology and lateral dimension, as shown in Fig. 1. Based on the TEM assay, lateral size of rGO in cell culture medium were mainly in the range of 50–700 nm(Fig. 1D), after sonication (20 kHz, 100 W, 5 min). AFM analysis showed that the thickness of rGO was less than 1 nm in the topographic height (Fig. 1E–H). rGO before sonication were larger in size and thicker compared to after sonication(Fig. S1).

### Cell viability

**Figure 2:**
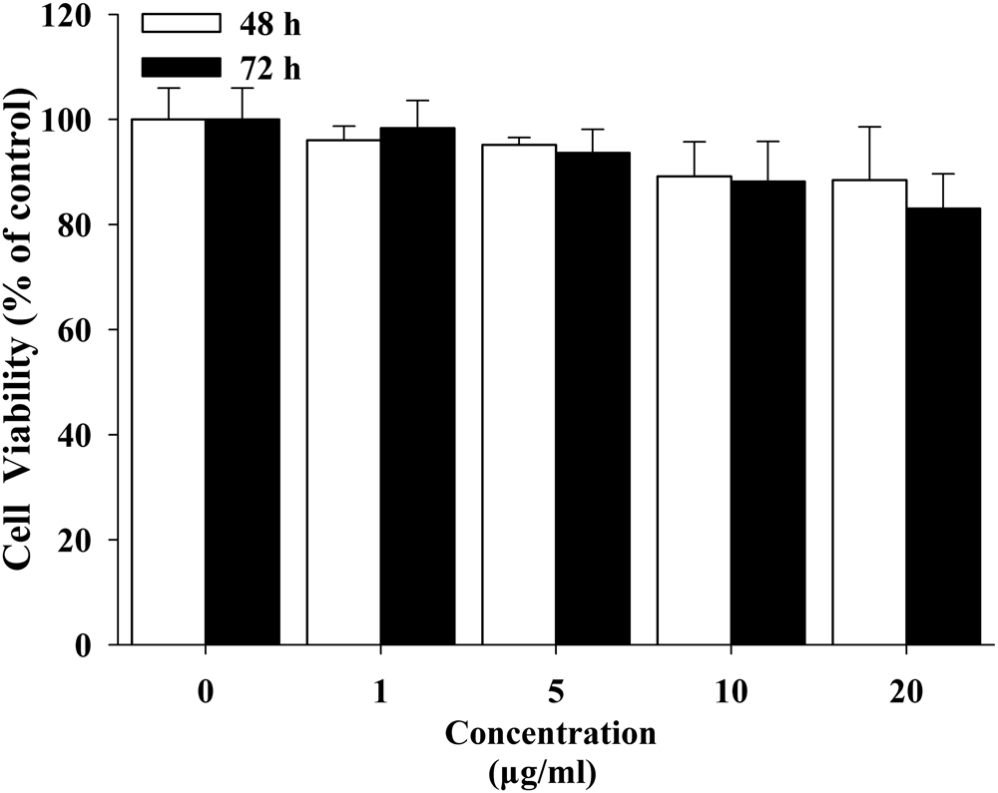
The viability of A549 after exposure to rGO with concentrations of 1–20 μg/ml for 48 h or 72 h. Cytotoxicity of rGO was assessed using the MTS assay and the values were given as the means ± SD (n = 3).

MTS assay was performed to investigate the effects of the rGO on the viability of A549 cells after 24 h and 48 h of rGO treatment. No obvious loss in viability had occurred from treatment with rGO at the tested concentrations ranging from 1 to 20 μg/ml compared to the control (Fig. 2), even at the dose of 20 μg/ml where the number of viable cells slightly reduced. This result indicated that the rGO concentrations in these experiments showed no suppressive effects on cell survival.

### Cell uptake

**Figure 3:**
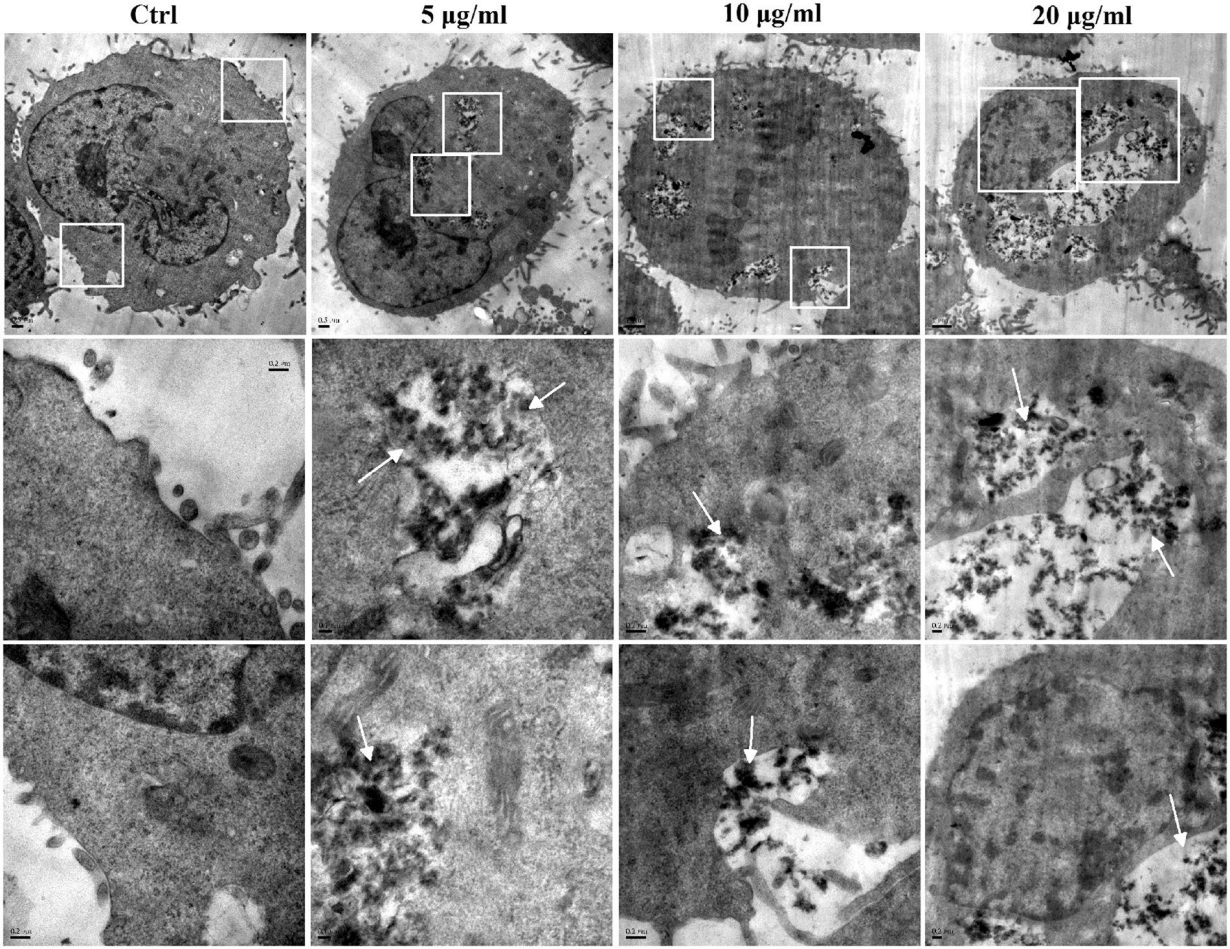
TEM micrographs of A549 after exposure to rGO at 5, 10 and 20 μg/ml for 48 h. The rGO aggregates were taken up by A549, but cell morphology upon rGO exposure was similar to control group. TEM micrographs on the bottom represent high-magnification images of the white marked box of the photos on top. The white arrows point to intracellular aggregation of rGO nanoplatelets.

The ultrathin sections of rGO-treated A549 cells was observed under TEM for the uptake of nanosheets. Firstly, we found numerous rGO nanosheets inside A549 cells (Fig. 3). rGO nanosheets were aggregated in the cytoplasm under TEM as shown in cell culture medium previously. The nanosheets with lateral dimension of approximately 100 to 300 nm can be observed. Nanosheets with lateral sizes over 400 nm were sparse. In contrast, these structures inside the cells were not observed in the control group. On some occasions rGO at the A549 cell surface led to the formation of membrane invaginations (Fig. 3). The images provided evidence that rGO aggregates accumulated inside A549 in the cytosol or enveloped within a membrane. The capability of internalization of graphene derivatives has been also detected in other cell lines previously^18,35^.

### Immunoelectron microscopic analysis

**Figure 4:**
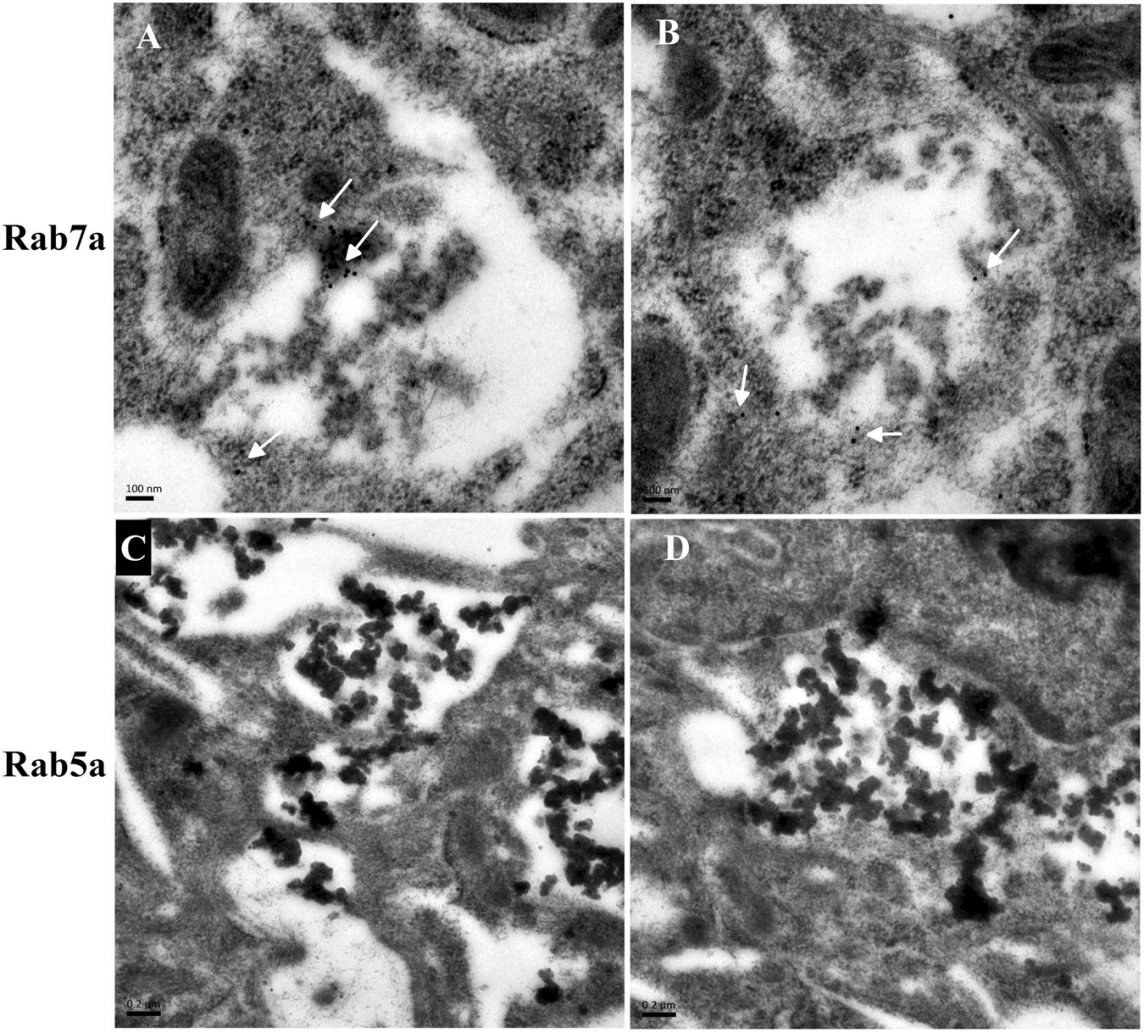
Ultrastructural localization of Rab7a and Rab5a in A549 after treatment with rGO. (**A**,**B**) TEM analysis revealed immunogold labeled Rab7a particles localized to vacuolar membranes in rGO-treated cells (shown with 15 nm gold particles, white arrows). **(C**,**D)** None or trace labeling of Rab5a (15 nm gold particles) in A549.

The rGO-teated ultrathin cell sections were processed through immunogold labeling experiments, where the Rab7a were observed at the membranes of the vacuolar containing rGO. Little or no Rab5a immunogold staining was observed in A549 cells incubated with rGO for 48 h. In Fig. 4A,B, the white arrows indicate the mmunogold-labeled Rab7a which is the late endosomal markers, and it is clearly embedded around the margins of rGO-induced vacuoles. Moreover, the early endosomal protein of Rab5a (Fig. 4C,D) was rarely associated with the vacuolar membranes. These findings reinforced rGO penetrated through the A549 cells membrane into the cytosol by endocytosis and located in late endosome.

### Cell wound healing assay

**Figure 5:**
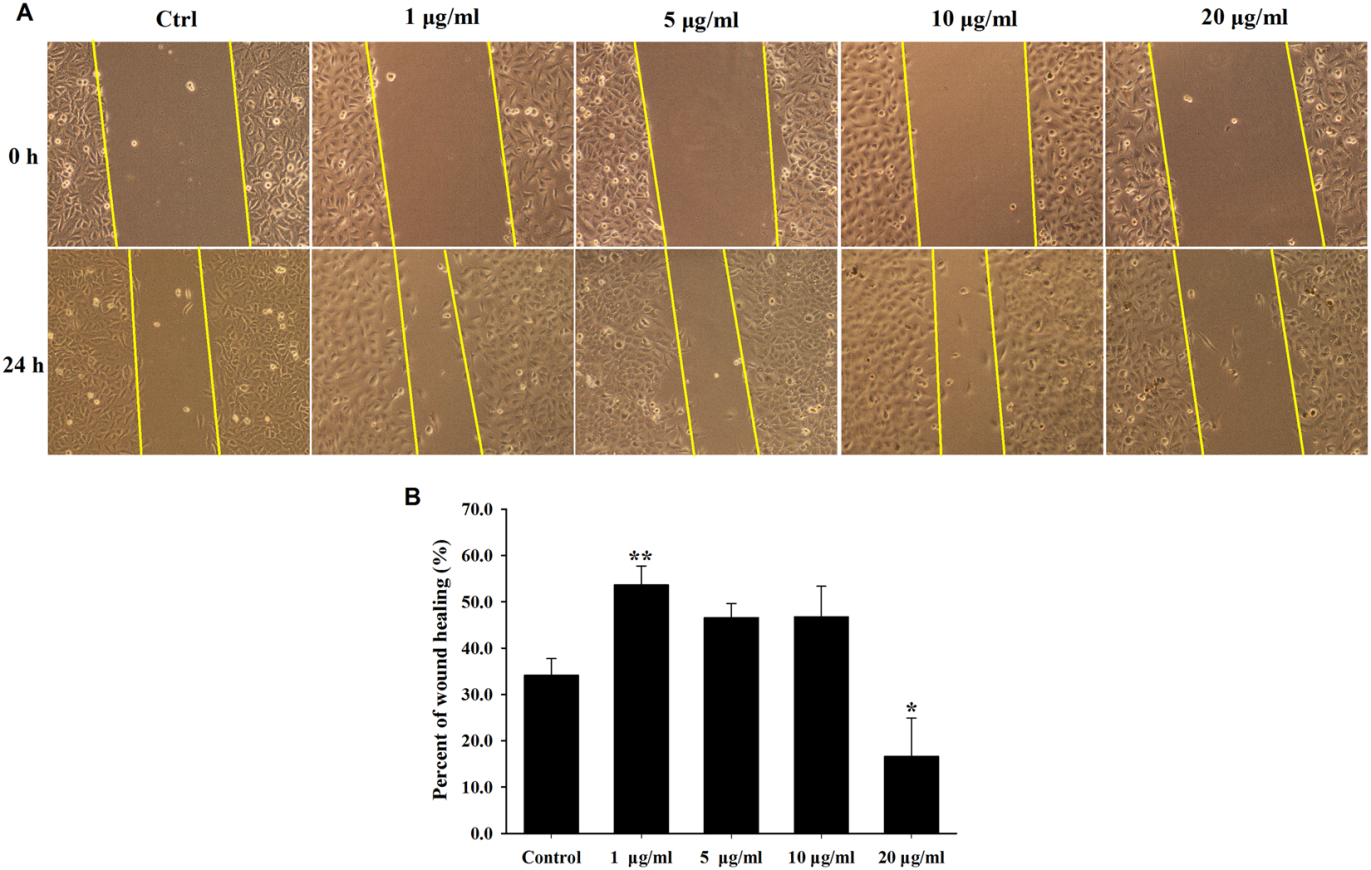
Effect of rGO on A549 migration into an artificially induced wound. (**A**) Representative images from the wound healing scratch assay at 0 and 24 h timepoints in the presence of rGO (0, 1, 5, 10 and 20 μg/ml). (**B**) The migratory level of A549 cells were quantified by the wound area closure, *indicates significant difference from control with P < 0.05, and values are the mean ± SD of three independent experiments.

The effect of rGO on A549 migration was assessed using wound healing assay across concentrations ranging from 1 to 20 μg/ml. The results showed an increased the wound closure of A549 cells in lower concentration rGO treatment (1, 5 and 10 μg/ml) for 48 h, whereas higher concentration treatment (20 μg/ml) inhibited A549 migration potential into an artificially induced wound when compared to the control group (Fig. 5A). The normalized wound healings are shown in Fig. 5B.

### The invasion of A549 cells

**Figure 6:**
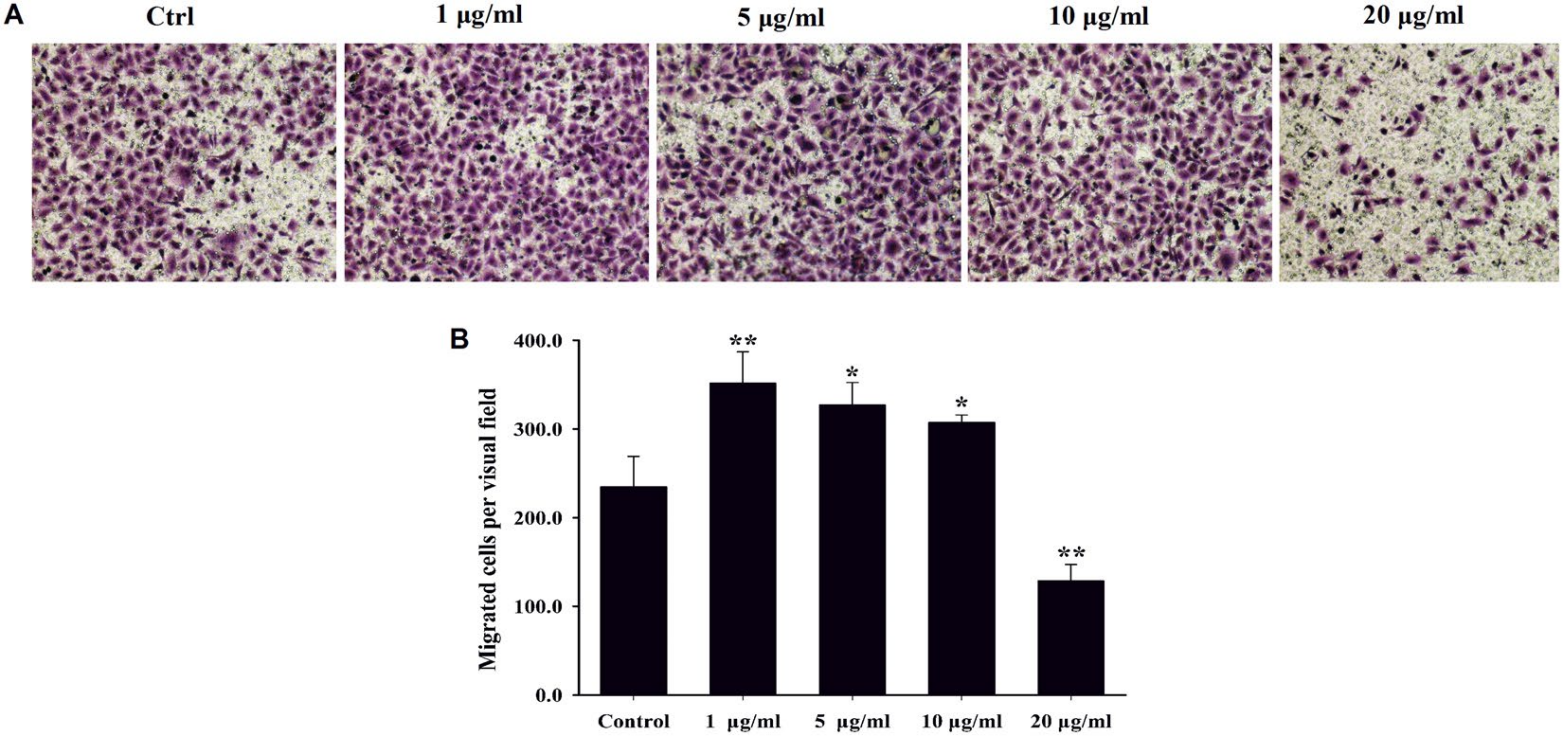
Migration of A549 cells after rGO treatment.(**A**) Cells were used in Transwell Matrigel invasion assay with the indicated dose of rGO for 48 h in the upper well, and then the cells were incubated for 24 h at 37 °C during the invasion assay. A549 Cells that invaded to the lower side of the membrane were fixed and stained with crystal violet. Representative optical photos are shown. (**B**) The invading cell numbers were quantified by counting. Values are the means ± SD from three independent experiments (**p < 0.01, *p < 0.05).

Transwell invasion assay was carried out to investigate invasion capacity which was important feature of malignant cells (Fig. 6). The Transwell chamber inserts were pre-coated with Matrigel, mimicking the extracellular matrix. In the invasion assays, the number of A549 cells in the lower concentration of rGO treatment group was significantly higher than that in the negative control group, while the number of cells in the higher concentration treatment group was significantly decreased compared with the negative control group. The results was similar to that of wound healing assay.

### The EMT-related gene expression levels

**Figure 7:**
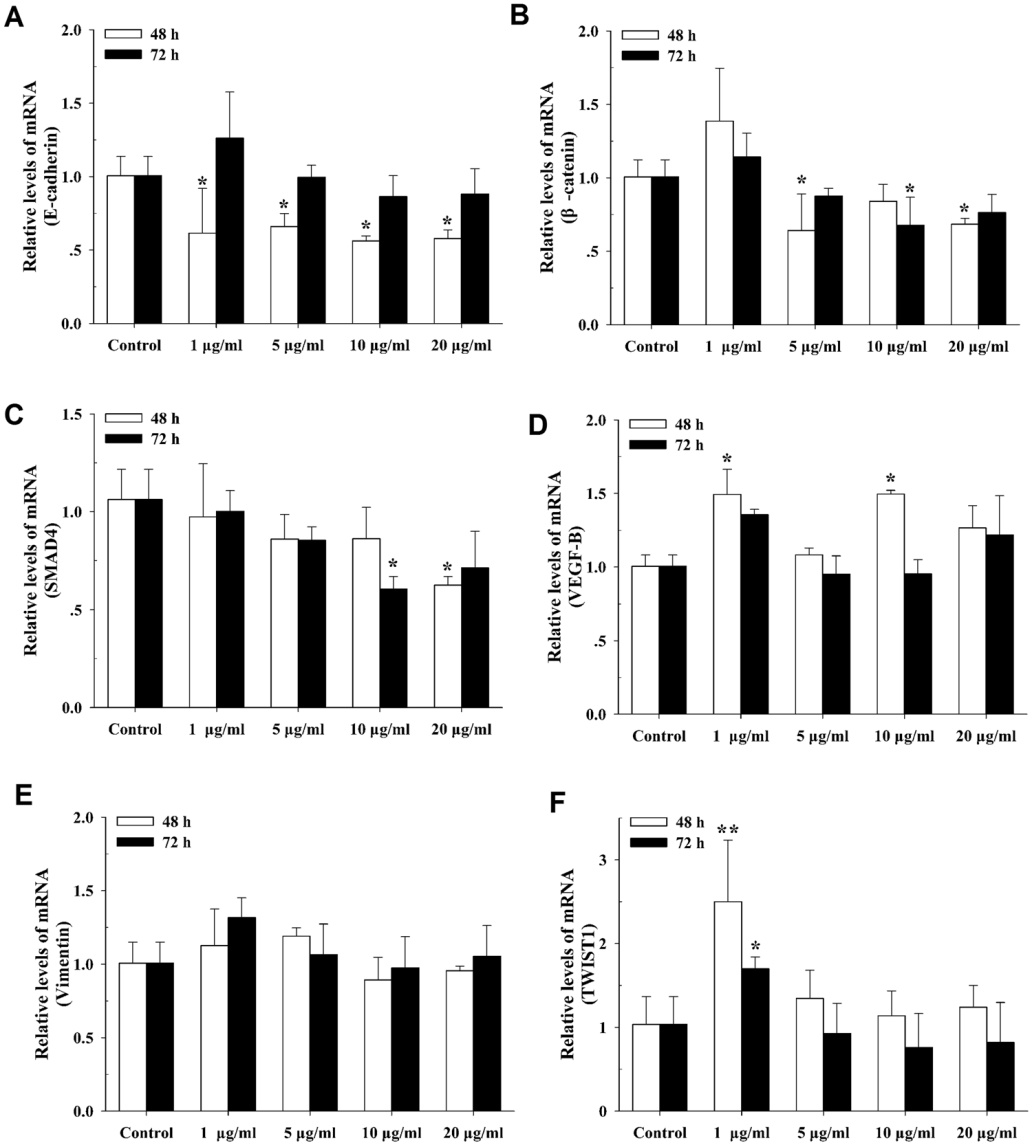
Expression of EMT-related genes after rGO treatment in A549. The relative expression levels of E-cadherin (**A**), β-catenin (**B**), Smad4 (**C**), VEGF-B (**D**), Vimentin (**E**) and TWIST1 (**F**) were measured by SYBR Green-based qRT-PCR using GAPDH as an internal reference. Relative mRNA amounts were quantified by the comparative Ct method (2^−△△Ct^). The values are presented as the mean ± SD of three independent experiments carried out in triplicate. **p < 0.01, *p < 0.05 compared to the control.

To further investigate the reason for introduction of EMT in A549 with the treatment of rGO, we examined the effects of rGO on the mRNA levels of EMT markers, including *E-cadherin*, *β-catenin*, *Smad4*, *VEGF-B*, *Vimentin* and *TWIST1*. As shown in Fig. 7, stimulation of A549 with rGO for 48 h resulted in a significant decrease in the expression of *E-cadherin*, *β-catenin* and *Smad4* genes. When the cells were incubated for 72 h with rGO at 10 μg/ml, the expression of *β-catenin* and *Smad4* were significantly down-regulated, but no significant alteration in the expression of *E-cadherin* was observed. In addition, *E-cadherin*, *β-catenin* and *Smad4* did not show any statistically significant differences between the groups of cells treated with 1, 5 and 20 μg/ml rGO for 72 h and the control group (Fig. 7A–C). After treatment with rGO for 48 h, significantly increased expression of *VEGF-B* in the group of cells treated with 1 and 10 μg/ml rGO had occured. While only the expression of *TWIST1* in the cells treated with 1 μg/ml rGO for 72 h was obviously enhanced (Fig. 7F), of *VEGF-B*, *Vimentin* and *TWIST1* genes with various concentrations remained unchanged.

### The EMT-related protein expression levels

**Figure 8:**
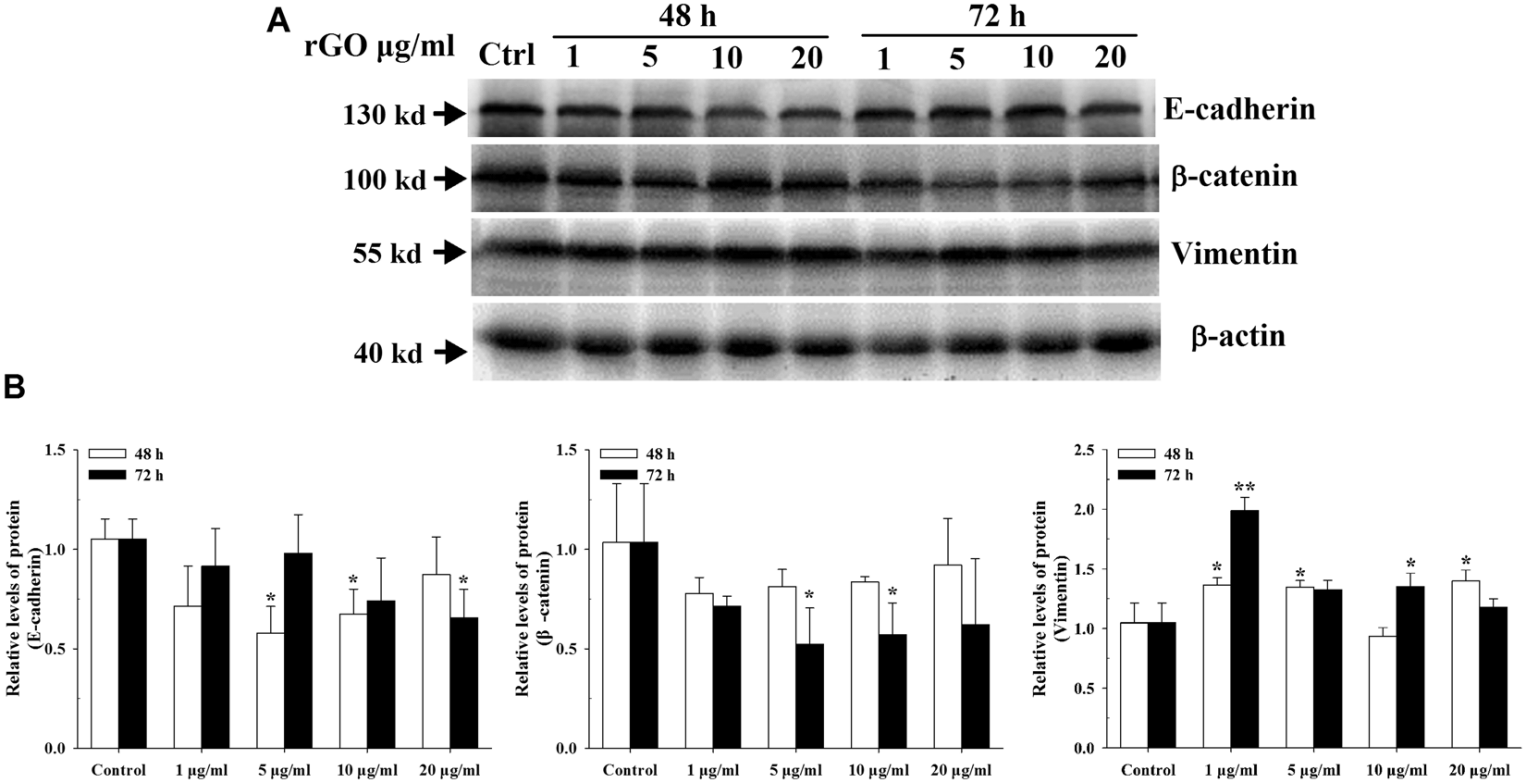
Western blotting of E-cadherin, β-catenin and Vimentin. A549 cells were treated with rGO 1, 5, 10 or 20 μg/ml for 48 or 72 hours and protein samples were evaluated to Western blotting assay. (**A**) Representative blots of E-cadherin, β-catenin and Vimentin in A549 cells. The grouping of blots images was from different parts of the same gel. The gels were cropped for conciseness. All gels were run simultaneouslyunder the same experimental conditions. Arrows show locations of molecular weight markers. (**B**)Bar graphs showing the relative levels of protein E-cadherin, β-catenin and Vimentin in A549 cells. Data are shown as the mean ± SD of three independent experiments. **p < 0.01, *p < 0.05 by one-way analysis of variance.

Regarding the protein expression of EMT markers, representative EMT markers (E-cadherin, β-catenin and Vimentin) were verified through western blotting. Figure 8 shows clearly E-cadherin and β-catenin protein levels were decreased in A549 cells after exposure to rGO. There was a 1.4-, 1.3-and 1.4-fold increase in Vimentin in A549 cells treated with rGO 1, 5 and 20 µg/ml respectively, for 48 hours. In addition, the ratio of Vimentin was markedly increased by 2.0-,and 1.4-fold in A549 cells treated with rGO 1 and 10 µg/ml, respectively for 72 hours.

## Discussion

Although impressive array of physicochemical features, the peculiar properties of graphene raise concerns about their potential health risks following occupational or environmental exposure. However, the cytotoxicity of graphene and the underlying toxicity mechanisms have not been well understood. In this paper, a range of 1–20 μg/ml rGO concentrations were chosen to treat A549 cells because this range was also used in previous studies for toxicity studies on graphene and its derivatives reported by other researchers^12^. Results from cell viability assay showed that rGO (1–20 μg/ml) has no significant cell proliferation inhibition. This result was comparatively consistent with the study of YanliChang *et al*. and Wendi Zhang *et al*., in which no obvious cytotoxicity were observed after treatment with 10–25 µg/ml graphene oxide (GO) for 24 h in A549 or mouse embryo fibroblasts cells^13,35^. With the exception of the research reporting the good biocompatibility of graphene, there is literature reporting that graphene-based materials has higher toxicity in cells and animals at various concentrations^34,36–39^. For example, Wang *et al*. found that graphene derivatives is toxic to human fibroblast cells at the concentration of 50 μg/mL and higher^40^. In a study by Lammel *et al*.^18^, in which GO has a dose-dependent toxic effect through plasma membrane damage, resulting in altered cell morphology and an augmented number of apoptotic cells. Jaworski *et al*.^41^studied the toxicity of both GO and rGO in U87 platelets and U118 glioma cells. These results indicated GO and rGO enter glioma cells and show dose-dependent toxicity. The graphene synthesis/film preparation and the testing models may contribute to the inconsistency.

Microscopic visualization of interactions between rGO and A549 cells showed that rGO could be taken up by cells. The rGO folds and aggregates after adding into the culture medium and the aggregates in cells and displayed distinguishable under TEM. Based on experimental observations in current study, rGO is swallowed by A549 cells via endocytosis and located in late endosomes and/or lysosomes, indicating a strong affinity with the cells. Previous study demonstrated that three GOs with different oxidation degrees can be internalized by mouse embryo fibroblasts^35^. Lammel *et al*. demonstrated that GO and carboxyl graphene (CXYG) nanoplatelets were able to penetrate through the plasma membrane, disrupte the phospholipid bilayer and increase intracellular reactive oxygen species (ROS) levels^18^. Sasidharan *et al*.^19^ reported that carboxyl functionalized graphene could be internalized by monkey kidney cells, while pristine graphene accumulated in the cell membrane instead of being internalized by the cells. These results contrast with the work of Chang *et al*. who reported m-GO (430 ± 300 nm) was not found entering the A549 cells^13^. We proposed that the internalization of nanosheets also may be influenced by the shape, size and functionalized groups of materials.

Accumulating evidence indicates that graphene and GO exposure induce pulmonary fibrosis in mice and rat models^22–24^. Recently, epithelial cells has been increasingly recognized as an important role in the pathogenesis of lung fibrosis. Epithelial injury can lead to EMT, which represents a gradual cellular transformation process in which the epithelial cells undergo transition to a mesenchymal phenotype, giving rise to fibroblasts and myofibroblasts generation^42^. Besides, previous studies have shown an important role of EMT in carbon nanotubes-mediated pulmonary fibrosis^30^. Based on these points, we further studied the activation of EMT induced by rGO in epithelial cells. In our study, wound healing scratch and Transwell invasion assay showed that lower concentration of rGO treatment could effectively enhance the A549 cell migratory and invasive capacity (Figs 5 and 6). On the contrary, exposure to higher concentrations of rGO (20 μg/ml) displayed a suppressive ability in the cells. We also found similar results in BEAS-2B cells which is lung/brunch normal epithelial cell line, lower dose of rGO treatmentpromoted the cell migration and invasion capacities, but higher concentrations inhibited the capacities(Figs S4 & S5).

Furthermore, the expression levels of the six genes (*E-cadherin*, *β-catenin*, *Smad4*, *VEGF-B*, *Vimentin* and *TWIST1*) involved in EMT were evaluated in rGO-induced A549. Our finding showed that, for epithelial markers (*E-cadherin*, *β-catenin* and *Smad4*), the relative mRNA expression levels were decreased after a 48 h treatment with different concentrations of rGO in A549 cells and BEAS-2B cells (Figs 7A–C & S6). E-cadherin down regulation was associated with EMT that accounted for increased invasion and metastasis during tumor progression^43^. The typical characteristic of the EMT process was the loss of intercellular adhesion with E-cadherin down regulation that generated a tight junction to connect adjacent cells^44^. Catenins, such as β- and γ-catenin, bind to the cytoplasmic portion of E-cadherin^45^. The membrane loss of β-catenin is responsible for promoting the invasive character of the malignant tumors, through the reduced cellular adhesion^46^. Smad4 could induce EMT via TGF-β/Smad4 signals as previously described^47^. EMT is also characterized by the up-regulation of mesenchymal markers (eg. Vimentin, Twist1 and VEGF-B). Vimentin functions as a positive regulator of EMT and upregulation of vimentin appears to be a prerequisite for EMT induction^48^. Twist1 is known as a crucial marker of EMT and thought to function primarily through E-cad repression^49^. Vascular endothelial growth factor (VEGF) was induced by exposure of retinal pigment epithelial cells to cigarette smoke extract that are involved in promoting EMT^50^. We have found that the expressions of Twist1 and VEGF-B genes were up-regulated by rGO, but there was no promotion effect of Vimentin gene (Fig. 7E). We also described the protein status of three EMT markers. Interestingly, we captured increment in protein but not RNA levels of Vimentin, suggesting posttranslational regulation of this adherens junction component after rGO induction. In addition, we observed the reductions of E-cadherin and β-catenin protein levels (Fig. 8). Our results revealed that rGO exposure promoted the protein expression of Vimentin, accompanied by a decreased expression of E-cadherin, which routinely used in standard analysis of EMT. The genes and proteins of EMT marker had been changed after the cells were exposed to 20 µg/ml rGO. Contrary to this, the migration and invasion abilities was decreased. We surmised that increased rGO uptake by the cell treatment with higher concentration exposure, possibly destroyed the cell structure and resulted in the dysfuntion of cell due to the loss of integrity (Fig. 3). Although it was not found that the cell viability was suppressed, some other cell functions maybe affected.

## Conclusions

**Figure 9:**
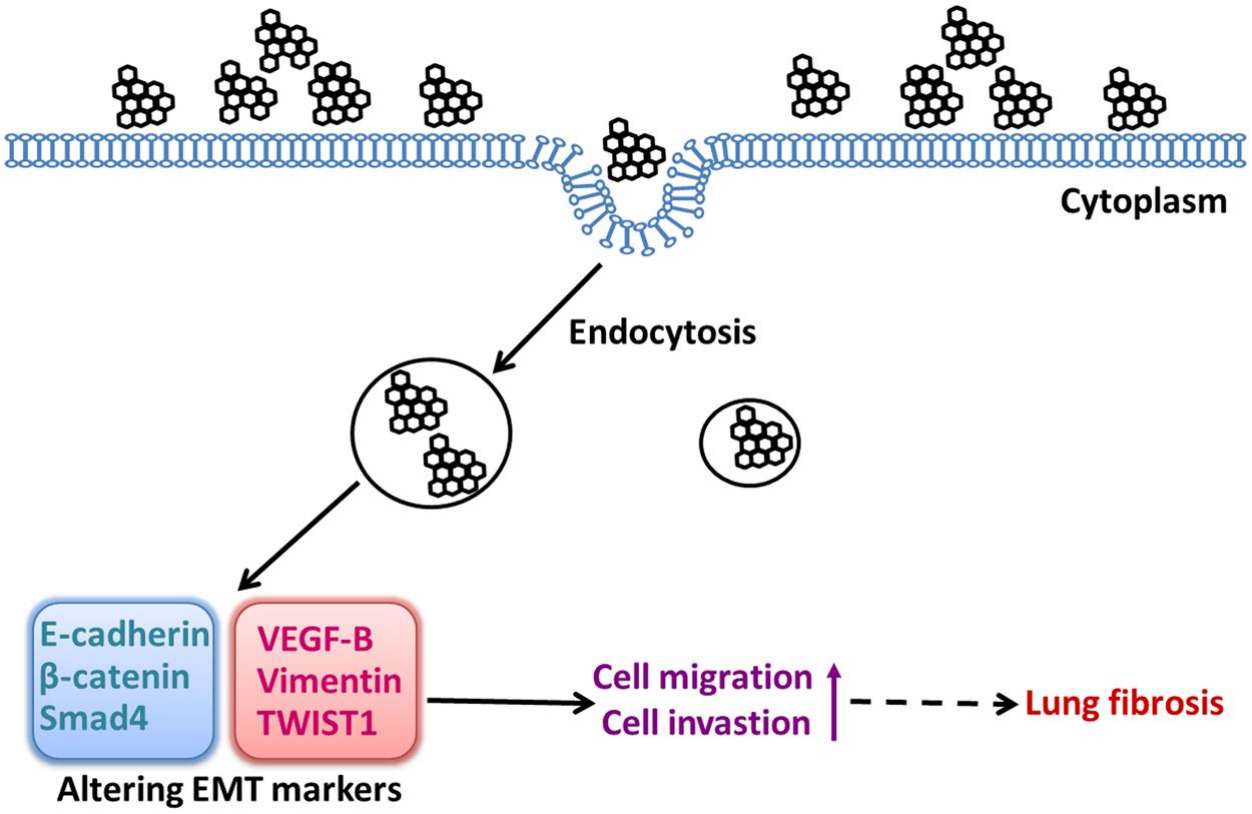
rGO triggered epithelial-mesenchymal transition in A549 cells.

In conclusion, this is the first study demonstrating that rGO, can induce EMT in an *in vitro* human model (A549 cells) after 48 h or 72 h incubation (Fig. 9). The rGO can be internalized by A549 with low cytotoxicity. Interestingly, lower concentration of rGO treatment can enhance cell invasive and migratory abilities, but higher concentration treatment inhibit this abilities. The reasons for this phenomenon maybe the structural damage of cells after rGO internalized by endocytosis. This study provides evidence of the EMT effect of rGO stimulation and points out the potential health risks associated with rGO. The findings should haveimportant implications for broadapplication of rGO inbiological applications. Doubtless, further investigations will be required to elucidate the underlying molecular signaling pathways associated with rGO-induced EMT.

## Materials and Methods

### Reagents and materials

rGO was purchased from the JCNANO Tech Co. Ltd (Nanjing, Jiangsu, China). Dulbecco’s Modified Eagle Medium/Nutrient Mixture F-12 (DME/F-12) and phosphate-buffered saline (PBS) solution were obtained from HyClone (Logan, Utah, USA). Fetal bovine serum (FBS)was from Gibco Invitrogen (CA, USA). 3-(4,5-dimethylthiazol-2-yl)-5-(3-carboxymethoxyphenyl)-2-(4-sulfophenyl)-2H-tetrazolium(MTS) was obtained from Promega (MO, USA). 24-well Transwell with 8-μm pore size polycarbonate inserts were purchased from Corning Incorporated (NY, USA). Trizol reagent was from Invitrogen Inc. (Carlsbad, CA, USA). The PrimeScript® RT Master Mix Perfect Real-Time kit and SYBR® Premix Ex TaqTM II kit were from Takara (Dalian, China). RIPA buffer and BCA protein assay kit were purchased from Thermo Scientific (Rockford, IL, USA). Monoclonal β-actin, polyclonal Rab5a, Rab7a, E-cadherin, β-catenin and Vimentin antibody were purchased from Proteintech (Rosemont, IL, USA). Goat anti-rabbit IgG conjugated with 15 nm gold particles were from Abcam (Cambridge, MA, USA).

### rGO characterizations

rGO sample was characterized by transmission electron microscopy (TEM) (H-7650, Hitachi, Japan) and atomic force microscopy (AFM, SPM-9600,Shimadzu, Japan). Commercial rGO was dispersed in ultra-pure water to prepare the stock solution (4.0 mg/mL). The stock solution was sonicated for 10 s, 16 times (40 kHz, 50 W) and prepared for TEM by drop casting the suspension onto a cleaned micasubstrate with 300 square mesh copper grids and dried at room temperature for 24 h.

### Cell culture and cell treatments

A549 cells were purchased from the Shanghai Cell Bank of Type Culture Collection (Shanghai, China). Cell culture and viability assay A549 cells were cultivated in DME/F12 medium supplemented with 10%(v/v)fetal bovine serumin the condition of 37 °C and 5% CO^2^. The stock solution of ultrasound-treatedrGO was diluted with DME/F12 culture medium to achieve the the working concentrations (0.5, 1, 5, 10 and 20 µg/ml) just before biological exposure.

### Cell proliferation assay

A549 cells were seeded in a 96-well plates at 9,000 cells/well. For proliferation assays, cell numbers were measured at 48 hours or 72 hours after treated with rGOin an indicated range of concentrations using a MTS Proliferation Assay Kitaccording to manufacturer’s instruction. Absorbance at 490 nm was determined by a multifunctional microplate reader (SpectraMaxM5, Molecular Devices, USA) and cell viability was calculated by the percentage of surviving cells compared with the control cells. The assays were performed as triplicate.

### Immunolabeling electron microscopy

A549 cells were washed with PBS and fixed with 4% paraformaldehyde for 4 h at 4 °C. Then, cells were fixed, dehydrated, embedded and mounted on formvar-coated nickel grids. For immunoelectron microscopy, the grids were incubated with anti-Rab5a and Rab7a (rabbit polyclonal; 1:50 dilution) as primary antibodies, followed by treatment with goat anti-rabbit 15 nm gold particles diluted 1:30 in PBS as secondary antibody. Grids were analyzed with a Hitachi H-7650 TEM after staining with uranyl acetate followed by Reynold’s lead citrate.

### Scratch wound migration assay

Cells were seeded in 12-well plates at a density of 4 × 10^5^cells/well. When cells reached 90% confluence, a scratch straight wounds was introduced with 10 µl pipette tips. Immediately following wounding, wells were washed three times with PBS and replaced with fresh medium. Cells were cultured in incubator for 24 h. The wound closure of each well was photographed by microscopy with a digital camera. The percentage of wound healing was quantified by the physical separation remaining between the initial wound areas using image j software. Data represent the mean of at least three independent experiments.

### Cell invasion assay

Cell invasion assays were done in 24-well transwell polycarbonate filters which were pre-coated with Matrigel (BD Biosciences). The cells in the medium with 1% FBS were seeded into the upper chambers of the transwells and 600 µl of DME/F12 containing 10% FBS was added to the lower chamber. After incubated for 24 h at 37 °C with 5% CO_2_, the transwell chambers were taken out and moved into wells containing 800 µl of methanol for 20 min. Penetrating cells were fixedf or 20 min at room temperature and then stained with 0.1% crystal violet for 30 min. For quantification, all of the cells that had penetrated onto the lower surface were examined under inverted light microscope (CKX41, Olympus, Tokyo, Japan). Four independent assays were performed and results are shown as the mean ± standard error of the mean (SD).

### Quantitative real-time polymerase chain reaction(qRT-PCR)

Total RNA from the A549 cells was isolated using Trizol reagent and reverse transcribed into cDNA using the PrimeScript® RT Master Mix Perfect Real-Time kit according to the manufacturer’s instructions. Thereafter, quantitative real-time PCR was performed on a Roche 4800 instrument. The sequences of the primers for *GAPDH*, *E-cadherin*, *β-catenin*, *Smad4*, *VEGF-B*, *Vimentin* and *TWIST1* were shown in Table 1. The mRNA expression of these genes was quantified with SYBR® Premix Ex TaqTM II kit. GAPDH was used as an endogenous control. The relative level of the mRNA expression was calculated with 2^−∆∆ct^method.

**Table 1:** Primer sequences for quantitative real-time PCRamplification.

Gene name	Accession number	Primer sequences used for qRT-PCR (5′ to 3′)
GAPDH	NM_002046.6	F: GAAGGTGAAGGTCGGAGTC
		R: GAAGATGGTGATGGGATTTC
E-cadherin	NM_001317185.1	F: CTGAGAACGAGGCTAACG
		R: GTCCACCATCATCATTCAATAT
β-catenin	NM_001904.3	F: CAAGTGGGTGGTATAGAGG
		R: GGATGGTGGGTGTAAGAG
Smad4	NM_005359.5	F: AAACCATCCAGCATCCAC
		R: GTTGGGAAAGTTGGCAGT
VEGF-B	NM_003377.4	F: GGAGATGTCCCTGGAAGAACA
		R: CTGGCTTCACAGCACTGTCC
Vimentin	NM_003380.4	F: TTGAACGCAAAGTGGAATC
		R: GGTCAGGCTTGGAAACATC
TWIST1	NM_000474.3	F: CGGGAGTCCGCAGTCTTA
		R: CTTGAGGGTCTGAATCTTGCT

### Western blotting

The cells in each group were lysed in 100 µl precooling RIPA lysis buffer containing phosphatase inhibitor Mix. The protein concentration wa sdetermined with a BCA protein assay kit according to the manufacturer’s instructions. Proteins were differentiated on SDS-polyacrylamide gel electrophoresis and then transferred onto a polyvinylidene difluoride (PVDF) membrane (Millipore Co., USA). Membrane was blocked with 5% nonfat milk in TBST and incubated at 4 °C over night with primary antibodies. Membranes were washed with TBST and incubated with applicable secondary antibodies in TBST. The bands were quantified and visualized by the Kodak 4000 MM ImageStation (Eastman Kodak, Rochester, NY, USA). The results were normalized to GAPDH as an internal control.

### Data analysis

The valueswere expressed as means ± standard deviation(SD) of at least three independent experiments performed in triplicate. SPSS 16.0 statistical software (SPSS, Inc., Chicago, USA) was used for statistical analysis. All statistical analyses were analyzed using one-way ANOVA with Tukey’ s post-hoc test. Statistical significance was set at *P < 0.05; **P < 0.01.

## Electronic supplementary material

Supplementary materials
