# Supplementary material for: Reduced graphene oxide triggered epithelial-mesenchymal transition in A549 cells
Source: Sci Rep. 2018 Oct 12;8:15188. doi: 10.1038/s41598-018-33414-x (PMC6185964; doi:10.1038/s41598-018-33414-x)
Supplement: Supplementary file 1 — Supplementary materials [file 41598_2018_33414_MOESM1_ESM.docx]

**Reduced graphene oxide triggered epithelial-mesenchymal transition in A549 cells**

Yanyan Liao^1,2,3,†^ , Weiyi Wang^1,2,4,†^, Xiaomei Huang^1,2,3^, Yongyan Sun^1,2,3^, Shen Tian^1,2^, Peng Cai^1,2,*^

1. Key Lab of Urban Environment and Health, Institute of Urban Environment, Chinese Academy of Sciences, Xiamen 361021, China. E-mail: yyliao@iue.ac.cn

2. Xiamen Key Laboratory of Physical Environment, Institute of Urban Environment, Chinese Academy of Sciences, Xiamen 361021, China.

3. College of Resources and Environment, University of Chinese Academy of Sciences, Beijing 100049, China

4. State Key Laboratory Breeding Base of Marine Genetic Resources, Key Laboratory of Marine Genetic Resources, Fujian Key Laboratory of Marine Genetic Resources, Fujian Collaborative Innovation Centre for Exploitation and Utilization of Marine Biological Resources, Third Institute of Oceanography, State Oceanic Administration, Xiamen 361005, China. E-mail: [wywang@tio.org.cn](mailto:wywang@tio.org.cn)

^†^ These authors contributed equally to this work.

^*^ Corresponding authors. E-mail: pcai@iue.ac.cn (Peng Cai)

**Supplementary Figure S1: Characterization of the rGO before sonication.** **(A, B, C, D)** TEM images. **(E, G)** AFM images. **(F, H)** AFM results of flake thickness for rGO.

^

^

**Supplementary Figure S2: The viability of A549 after exposure to rGO with concentrations of 1-20 μg/ml for 48 h.** Cytotoxicity of rGO was assessed using the MTS assay. Paclitaxel and hydrogen peroxide treatment group were as positive controls. The values were given as the means ±SD (n=3). ** indicates signiﬁcant difference from control with P < 0.01.

^

^

**Supplementary Figure S3: Immunoelectron microscopy showed no immunolabeling were detected in negative controls which were obtained by omitting the primary antibody.**

^

^

**Supplementary Figure S4: Eﬀect of rGO on BEAS-2B migration into an artiﬁcially induced wound.**

**(A)** Representative images from the wound healing scratch assay at 0 and 24h timepoints in the presence of rGO (0, 1, 5, 10 and 20 μg/ml). **(B)** The migratory level of BEAS-2B cells were quantiﬁed by the wound area closure, * indicates signiﬁcant difference from control with P < 0.05, and values are the mean ± SD of three inde pendent experiments.

**

**

**Supplementary Figure S5: Migration of BEAS-2B cells after rGO treatment. (A)** Cells were used in Transwell Matrigel invasion assay with the indicated dose of rGO for 48 h in the upper well, and then the cells were incubated for 24 h at 37°C during the invasion assay. BEAS-2B Cells that invaded to the lower side of the membrane were fixed and stained with crystal violet. Representative optical photos are shown. **(B)** The invading cell numbers were quantified by counting. Values are the means ±SD from three independent experiments (**p< 0.01, *p<0.05).





**Supplementary Figure S6: Expression of EMT-related genes after rGO treatment in BEAS-2B.** The relative expression levels of E-cadherin **(A)**, β-catenin **(B)**, Smad4 **(C)**, VEGF-B **(D)**, Vimentin **(E)** and TWIST1 **(F)** were measured by SYBR Green-based qRT-PCR using GAPDH as an internal reference. Relative mRNA amounts were quantiﬁed by the comparative Ct method (2-△△Ct). The values are presented as the mean ± SD of three independent experiments carried out in triplicate. *p<0.05 compared to the control.





**Supplementary Figure S7: The original uncropped and unedited pictures of the western blot.** A549 cells were treated with rGO 1, 5 10 or 20 μg/ml for 48 or 72 hours and protein samples were evaluated to Western blotting assay. The last two lanes are for other experiment.
